# Metformin Can Enhance the Inhibitory Effect of Olaparib in Bladder Cancer Cells

**DOI:** 10.1155/2022/5709259

**Published:** 2022-06-24

**Authors:** Bao-Jin Chi, Yao Sun, Ling-Li Quan, Jin-Tao Zhao, Bo Wei, Shu-Qiu Wang

**Affiliations:** ^1^Department of Urology, The First Affiliated Hospital of Jiamusi University, China; ^2^Department of Vascular Surgery, The First Affiliated Hospital of Jiamusi University, China; ^3^Pulmonary and Critical Care Medicine, The Affiliated Zhuzhou Hospital Xiangya Medical College CSU, China; ^4^Department of Gastroenterology, The First Affiliated Hospital of Jiamusi University, China; ^5^Basic Medical College, Jiamusi University, China

## Abstract

**Background:**

Bladder cancer is a common urinary system tumor. In the treatment of clinical patients, it is particularly important to find an effective treatment method to inhibit tumor growth. The world's first PARP inhibitor olaparib is mainly used for the treatment of BRCA1/BRCA2 mutated tumors. Metformin, an antidiabetic drug, has been reported to reduce cancer incidence in humans and improve survival in cancer patients.

**Methods:**

Cell viability and proliferation were detected by CCK-8 assay and colony formation assay; cell apoptosis was detected by flow cytometry; cell migration and invasion abilities were detected by scratch assay and Transwell assay; STAT3/C-MYC signaling pathway protein were detected by western blotting.

**Results:**

Olaparib combined with metformin has better effects on the proliferation, clone formation, migration, invasion, and apoptosis of bladder cancer cells than single drug, indicating that metformin can enhance the inhibitory effect of olaparib on tumor growth and regulate the expression of STAT3/C-MYC signaling pathway proteins.

**Conclusion:**

The results of this study showed that metformin could significantly enhance the antitumor effect of olaparib on bladder cancer cells, and these effects were mediated by downregulating STAT3/C-MYC signaling pathway proteins. This finding may have potential clinical application in the treatment of bladder cancer.

## 1. Introduction

Bladder cancer is the tumor with the highest incidence in the urinary system, and its incidence rate ranks 10th among all tumors. In 2020, there were about 580,000 new cases of bladder cancer worldwide, and the incidence and mortality of men were about 3 times that of women [1]. In China, bladder cancer has been on the rise in recent years. Bladder cancer patients in China increased from 55,000 in 2012 to more than 80,000 new cases in 2015 [2].

In 2014, the world's first PARP inhibitor, olaparib, was approved by the U.S. Food and Drug Administration (FDA) for the treatment of ovarian cancer. PARP inhibitors have been approved by the FDA for ovarian cancer, breast cancer, prostate cancer, and pancreatic cancer [3–5], its role in other tumors has also been gradually discovered, and it is expected to become a broad-spectrum antitumor drug. A variety of drugs are currently being used to study whether they can enhance the efficacy of olaparib in cancer treatment. Studies have shown that abiraterone, foretinib, PHA6657, and other drugs in combination with olaparib have synergistic effects when they act on tumor cells [6–11].

Metformin has been used clinically for more than 50 years and is currently one of the most widely used oral hypoglycemic agents in the world. In recent years, studies have shown that metformin has an inhibitory effect on bladder cancer and can improve the prognosis of bladder cancer patients [12, 13]. Studies have shown that metformin exerts anticancer effects through the following molecular mechanisms: activating the AMPK/MTOR signaling pathway and inhibiting the STAT3 signaling pathway [14–16].

In addition, metformin combined with gefitinib, pirarubicin, cisplatin, vitamin D3, and panobinostat can enhance the tumor suppressor effect [17–21]. Therefore, this study explored whether metformin combined with olaparib could enhance the tumor suppressor effect on bladder cancer cells.

## 2. Materials and Methods

### 2.1. Cell Lines and Cell Culture

Bladder cancer cell lines 5637 and BIU-87 were purchased from the Cell Collection Committee of the Chinese Academy of Sciences. The cells were inoculated in DMEM medium containing 10% fetal bovine serum, 1% penicillin, and streptomycin and placed in a 37°C, 5% CO_2_, and saturated humidity incubator for routine culture, and cells in logarithmic growth phase were taken for experiments.

### 2.2. CCK8 Assays

The cells in the logarithmic growth phase were routinely digested, centrifuged and made into a single cell suspension, and seeded in a 96-well plate at 2000 cells/well. After the cells adhered, 200 *μ*L of medium containing different concentrations of metformin (0, 2.5, 5, 10, and 20 mM) and olaparib (0, 5, 10, 20, and 40 *μ*M) was added; each concentration group was set 3 duplicate holes. After 24 hours of drug intervention, 100 *μ*L CCK8 (1 : 10) dilution solution was added to each well, and after 2-4 h of conventional culture, the absorbance value (OD value) of each well at a wavelength of 450 nm was detected by a microplate reader.

### 2.3. Clone Formation Assays

The cells were digested to prepare a cell suspension, and the seeding condition was to inoculate 500 *μ*L of the cell suspension containing 6000 cells per well. After 24 h, the drug was added, and 500 *μ*L of the culture solution was added to each well of the corresponding concentration of the drug. After about 5-7 days, the medium and drugs in the 6-well plate were removed, the remaining dead cells were washed with PBS externally, and 500 *μ*L of 4% paraformaldehyde solution was added to each well to dehydrate the cells to achieve the purpose of fixing cells. Residual paraformaldehyde was washed with PBS. The clone formation pictures were detected by microscope.

### 2.4. Scratch Assays

Take bladder cancer cells in good growth condition, transfer the cells into a 6-well plate, and transfer the culture plate to a 37°C incubator to culture until the cells are confluent. A 200 *μ*L sterile pipette tip was used to be perpendicular to the culture plate; make a straight line scratch in each well. Cell debris was washed away with PBS. After treating the cells with different concentrations for 48 h, the images were observed and collected under an inverted microscope. The migration percentage was calculated according to the following formula: (1 − distance after scratching for 48 h/initial distance of scratching) × 100% = migration percentage.

### 2.5. Transwell Assays

The cells were digested and centrifuged, and the cells were resuspended in serum-free DMEM. Gently add 700 mL of complete medium to the lower chamber of the transwell 24-well plate to avoid air bubbles. 200 *μ*L of serum-free cell suspension containing 4000 cells was slowly added to the upper chamber with a pipette and placed in the incubator for 48 h. Crystal violet staining soaking chamber for 30 min, and use a cotton swab to remove cells that did not migrate successfully from the upper chamber. The chamber was taken out and washed with PBS, dried, photographed with a microscope, and counted.

### 2.6. Apoptosis Detection by Flow Cytometry

After the cells were cultured in the medium containing different concentrations of drugs for 48 h, the cells were collected and made into a single cell suspension. The cells were washed twice with PBS precooled at 4°C, and the cells were resuspended in 250 *μ*L of binding buffer and adjusted the concentration of about 1 × 10^6^ cells/mL. Take 100 *μ*L of cell suspension, add 5 *μ*L Annexin V-FITC and 10 *μ*L l PI solution in sequence, mix well, and incubate at room temperature for 15 minutes in the dark. Add 400 *μ*L PBS for flow cytometry analysis.

### 2.7. Glucose Kit and Lactate Assay

5637 and BIU-87 cells were taken and seeded in 6-well plates at a density of 2 × 10^5^ per well. After 24 h of cell adherent growth, the drug-containing medium was changed. The medium was used as the control group, and the metformin (10 mM), olaparib (20 *μ*M), and their combination groups were used as the experimental group. After 24 h of drug action, the medium was collected in a 2 mL tube, and glucose kit and lactate assay kit were used to detect the glucose content and lactate production in the medium.

### 2.8. Western Blot

Total protein was extracted with RIPA protein lysate and PMSF, quantified by Bradford method, mixed with 6 × loading buffer and boiled for 5 minutes to denature the protein. 30 *μ*g of protein was electrophoresed on 12% SDS-PAGE for 2 hours and transferred to PVDF membrane. Block for 1 h in 5% nonfat milk in TBST, add the corresponding primary antibody, and incubate overnight at 4°C. After washing, secondary antibodies were added and incubated at room temperature for 2 h. After 3 washings with TBST, ECL development was performed, and the images were collected and analyzed using Image Lab software (Bio-Rad).

### 2.9. In Vivo Tumorigenesis

Select 6-week-old female nude mice, weighing 18 ± 2 g. After digestion, the cells were centrifuged at 1000 rpm and counted by microscope, and the corresponding volume of cell suspension was measured by subcutaneous injection of 2 × 10^6^ cells. After subcutaneous transplantation of nude mice, the growth of nude mice and tumor were observed every week. When the diameter of transplanted tumor was about 4 mm, the drug was administered intraperitoneally every 4 days. After 4 weeks, the serum of tail vein of nude mice was taken, the animals were killed, and the tumor tissue was taken. Measure the tumor size with vernier caliper and calculate the tumor volume formula: volume = 0.5 × length × width × width.

### 2.10. Statistical Analysis

All experiments were repeated 3 times, and measurement data were expressed as “mean ± standard deviation”. SPSS 20.0 software was used for statistical analysis, and *t*-test was used for comparison between the two groups. *P* < 0.05 indicated statistical significance.

## 3. Result

### 3.1. Metformin, Olaparib, and a Dual-Drug Combination Inhibit Proliferation of Bladder Cancer Cells

**Figure 1:**
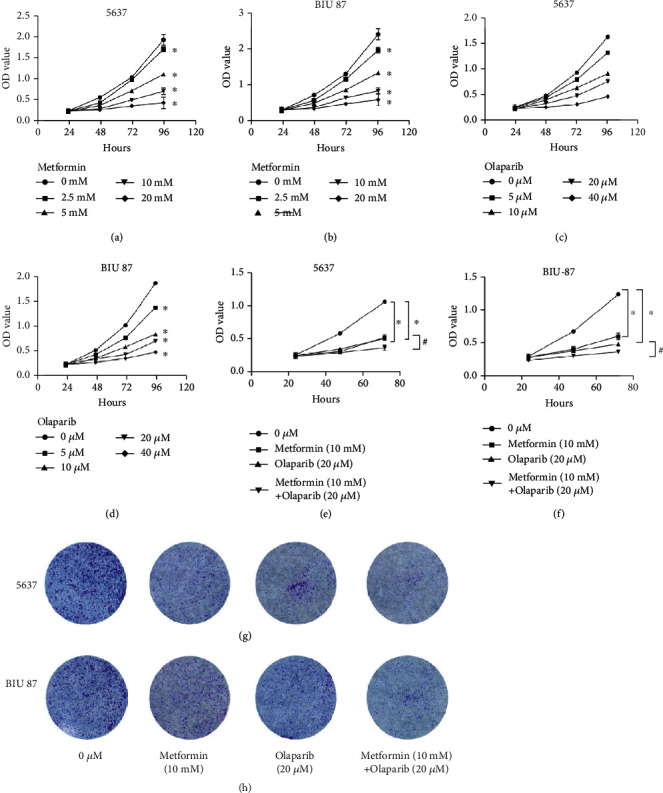
Metformin, olaparib, and a dual-drug combination inhibit proliferation of bladder cancer cells. (a and b) CCK8 assay showed that different concentrations of metformin had an effect on the proliferation of cells in 5637 and BIU87 cells. Data represent mean ± SD. ^∗^*P* < 0.05 compared with control. (c and d) CCK8 assay showed that different concentrations of olaparib had an effect on the proliferation of cells in 5637 and BIU87 cells. Data represent mean ± SD. ^∗^*P* < 0.05 compared with control. (e and f) CCK8 assay showed that a dual-drug combination metformin and olaparib inhibit proliferation of bladder cancer cells more effective. (g and h) Clone formation assay showed that a dual-drug combination metformin and olaparib inhibits proliferation of bladder cancer cells more effective. Data represent mean ± SD. ^∗^*P* < 0.05 compared with negative control. ^**#**^*P* < 0.05 compared with metformin or olaparib.

The CCK8 results showed that the inhibition rate of metformin and olaparib alone on 5637 and BIU-87 increased with time, and the inhibition rate of metformin (10 mM) and olaparib (20 *μ*M) combined was significantly higher than that of the two drugs alone (Figures 1(a)–1(d)) and was greater than the inhibition rate when used alone (Figures 1(e) and 1(f)). The CI of two drugs combined was calculated by calculyn1 software. The results showed that the CU of the two drugs in different effects (i.e., cell growth inhibition rate in combination) was less than 1, indicating that the combination of metformin and olaparib showed synergistic effect. It is suggested that metformin enhances the proliferation-inhibitory effect of olaparib on bladder cancer cells. After treatment with metformin (10 mM) and olaparib (20 *μ*M) alone, the colony formation of 5637 and BIU-87 cells was reduced, and the colony formation of 5637 and BIU-87 cells was more reduced after metformin (10 mM) and olaparib (20 *μ*M) combined treatment (*P* < 0.01) (Figures 1(g) and 1(h)). It was shown that the colony formation of both cells was significantly reduced due to the combination of these two drugs.

### 3.2. Effects of Metformin, Olaparib, and Dual-Drug Combination on Migration, Invasion, and Apoptosis of Bladder Cancer Cells

**Figure 2:**
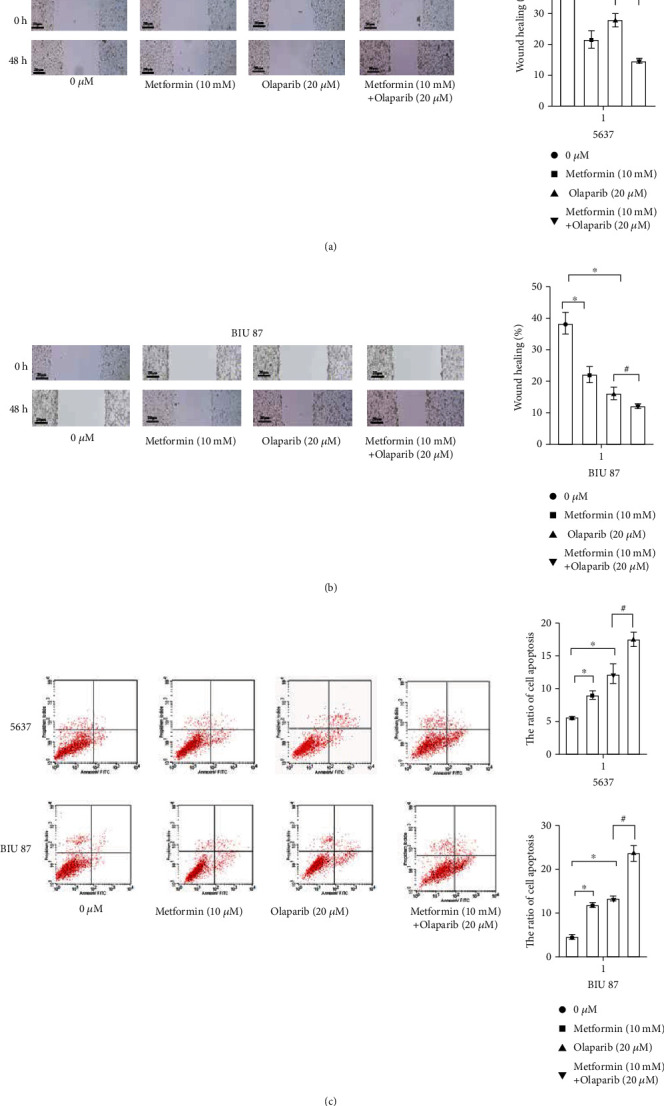
Effects of metformin, olaparib, and dual-drug combination on migration, invasion, and apoptosis of bladder cancer cells. (a and b) Wound healing showed that combination of metformin and olaparib significantly suppressed migration in 5637 and BIU87 cells. (c) The ratio of cell apoptosis was significantly increased in metformin and olaparib, compared with metformin and olaparib alone group. Data represent mean ± SD. ^∗^*P* < 0.05 compared with negative control. ^**#**^*P* < 0.05 compared with metformin or olaparib.

After treatment with olaparib and metformin alone, the inhibitor of wound healing in 5637 and BIU-87 cells was reduced, and the inhibitor of wound healing in 5637 and BIU-87 cells was more reduced after metformin and olaparib combined treatment (*P* < 0.01) (Figures 2(a) and 2(b)). It was shown that the migration number of both cells was significantly reduced due to the combination of these two drugs.

In order to confirm that the synergistic effect of metformin and olaparib is related to apoptosis, flow cytometry was used to detect the changes of apoptosis induced by metformin and olaparib alone or in combination. The results showed that compared with the control group, the ratio of cell apoptosis in metformin and olaparib alone group was significantly increased. The ratio of cell apoptosis was significantly increased in metformin and olaparib, compared with the metformin and olaparib alone group (Figure 2(c)), suggesting that the combination of metformin and olaparib could induce the increase of apoptosis in 5637 cells.

### 3.3. Effects of Metformin, Olaparib, and Dual-Drug Combination on Glucose Uptake and Lactate Production in Bladder Cancer Cells

**Figure 3:**
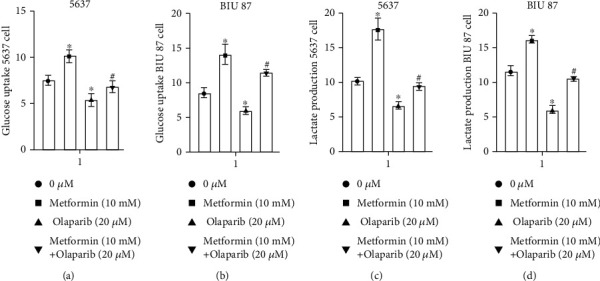
Effects of metformin, olaparib, and dual-drug combination on glucose uptake and lactate production in bladder cancer cells. (a–d) Compared with the control group, the glucose consumption and lactate production in the metformin group were significantly increased, while the glucose consumption and lactate production in the olaparib group were significantly decreased. Compared with the metformin group, the glucose consumption and lactate production in the two-drug combination group were significantly reduced. Data represent mean ± SD. ^∗^*P* < 0.05 compared with negative control. ^**#**^*P* < 0.05 compared with metformin or olaparib.

5637 and BIU-87 cells were treated with 10 mM metformin or olaparib (20 *μ*M) alone or in combination for 24 h, and the contents of glucose and lactate in the medium were detected. The results showed that compared with the control group, the glucose consumption and lactate production in the metformin group were significantly increased, while the glucose consumption and lactate production in the olaparib group were significantly decreased. Compared with the metformin group, the glucose consumption and lactate production in the two-drug combination group were significantly reduced (Figures 3(a)–3(d)), indicating that olaparib can reduce the accumulation of lactate caused by metformin.

### 3.4. Metformin Combined with Olaparib Induces a Decrease in the Expression of STAT3/C-MYC Pathway Proteins

**Figure 4:**
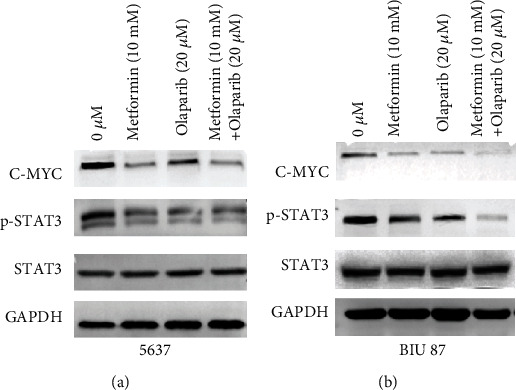
Metformin combined with olaparib induces a decrease in the expression of STAT3/C-MYC pathway proteins. (a and b) Western blot analysis of the expression levels of C-Myc, STAT3, and p-STAT3 which were treated negative control (NC) or metformin or olaparib or metformin combined with olaparib in 5637 and BIU87 cells. Data represent mean ± SD. ^∗^*P* < 0.05 compared with negative control. ^**#**^*P* < 0.05 compared with metformin or olaparib.

In order to further study whether the inhibition of cell proliferation induced by the combination of the two drugs is related to the changes in the expression of proliferation-related proteins, Western blot was used to detect the effects of metformin and olaparib alone and in combination on the expression of cell proliferation-related proteins. Compared with the control group, the expression levels of p-STAT3, STAT3, and C-MYC proteins in the cells of the metformin and olaparib alone group decreased (Figures 4(a) and 4(b)). The expression level was lower than that of the single-use group. It shows that the combination of metformin and olaparib reduces the proliferation of 5637 and BIU-87 cells, which may be related to the downregulation of p-STAT3, STAT3, and C-MYC protein expressions.

**Figure 5:**
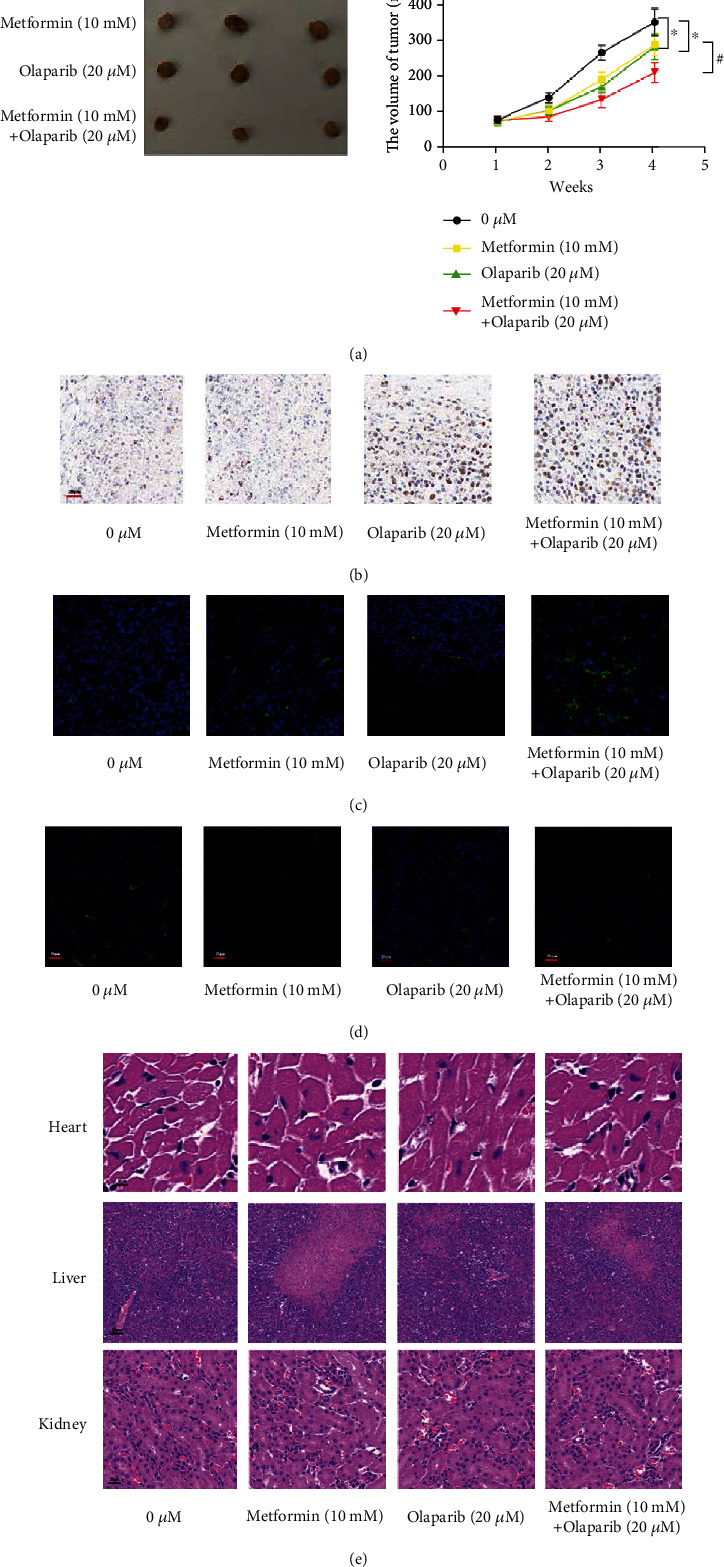
In vivo tumor, metformin combined with olaparib inhibits proliferation and promotes apoptosis. (a) Compared with the control group, the tumor formation volume was reduced. The tumor volume of 5637 cells was significantly reduced when metformin was combined with olaparib. (b) Ki67 result showed that after treatment with olaparib and metformin alone, the proliferation of tumor was more reduced after metformin and olaparib combined treatment. (c) Tunnel stain showed that after treatment with olaparib and metformin alone, the apoptosis of tumor was more increased after metformin and olaparib combined treatment. (d) Immunofluorescence result showed that after treatment with olaparib and metformin alone, p-STAT3 and C-MYC were reduced, and p-STAT3 and C-MYC were more reduced after metformin and olaparib combined treatment. (e) HE staining showed that after treatment with olaparib and metformin alone or metformin and olaparib combined treatment, there are no obvious abnormal changes in the heart, liver, and kidney, indicating that it has less toxicity to the heart, liver, and kidney. Data represent mean ± SD. ^∗^*P* < 0.05 compared with negative control. ^**#**^*P* < 0.05 compared with metformin or olaparib.

In vivo tumor formation experiments showed that after 5637 cells were treated with metformin or olaparib alone, compared with the control group, the tumor formation volume was reduced. The tumor volume of 5637 cells was significantly reduced when metformin was combined with olaparib (Figure 5(a)), Ki67 result showed that after treatment with olaparib and metformin alone, the proliferation of tumor was more reduced after metformin and olaparib combined treatment (Figure 5(b)). Tunnel stain showed that after treatment with olaparib and metformin alone, the apoptosis of tumor was more increased after metformin and olaparib combined treatment (Figure 5(c)). Immunofluorescence result showed that after treatment with olaparib and metformin alone, p-STAT3 and C-MYC were reduced, and p-STAT3 and C-MYC were more reduced after metformin and olaparib combined treatment (Figure 5(d)). HE staining showed that after treatment with olaparib and metformin alone or metformin and olaparib combined treatment, there are no obvious abnormal changes in the heart, liver, and kidney, indicating that it has less toxicity to the heart, liver, and kidney (Figure 5(e)).

## 4. Discussion

Bladder cancer is one of the most common malignant tumors in the urinary system. At present, the treatment of bladder cancer is mostly a combination of surgery and chemotherapy. The most effective way to prevent postoperative recurrence of bladder cancer patients is to supplement the intravesical infusion of immunosuppressants or chemotherapy drugs after surgery, but the recurrence rate of the tumor after surgery is as high as 60-70% [22, 23]. The severe side effects limit its clinical application. Therefore, finding new drug treatments and treatment methods is clinically important key issues that need to be addressed urgently.

The main mechanism of action of metformin is that it first inhibits gluconeogenesis by activating AMPKA and at the same time inhibits the expression of its downstream important signaling molecule mTOR [24, 25], thereby inhibiting the expression of protein. In the process of inhibiting tumor proliferation, this pathway molecule often also plays an important role. Numerous studies have confirmed that metformin has an inhibitory effect on a variety of tumors [26–28]. Recent clinical studies on bladder cancer show that the risk of tumor recurrence in bladder cancer patients who take metformin to lower blood sugar is significantly reduced [29, 30], suggesting that metformin has a certain degree of protection for bladder cancer patients and may enhance the efficacy of chemotherapy drugs.

In this study, bladder cancer cell lines 5637 and BIU-87 were used as the research object to detect whether metformin could enhance the sensitivity of bladder cancer cells to olaparib-induced apoptosis and to explore its possible molecular mechanism.

The results of this study confirmed that metformin can significantly increase the sensitivity of bladder cancer 5637 cells to olaparib, suggesting that metformin can be used as a sensitizer for olaparib in combination with olaparib for the treatment of bladder cancer. The combination of olaparib and metformin can jointly inhibit the PI3K/Akt/mTOR signaling pathway and exert a synergistic antitumor effect, which provides an experimental basis for the combination of metformin and olaparib in the treatment of bladder cancer.

## Data Availability

All data generated or analyzed during this study are included.
